# Comparative Quality Evaluation of Marketed Cefixime Trihydrate Tablet Brands Available in Bangladesh

**DOI:** 10.1155/tswj/4894162

**Published:** 2026-10-26

**Authors:** Ariful Islam, Shahriar Alam Shanto, Sumaiya Akter, Antara Nashrah, Mashiwat Mahboub, Zannatul Ferdous Fariha, Afia Anjum, Ferdous Khan, Md Hasanuzzaman Shohag

**Affiliations:** ^1^ Department of Pharmaceutical Sciences School of Health & Life Sciences North South University Dhaka Bangladesh northsouth.edu

**Keywords:** antibiotic resistance, postmarket surveillance, RP-HPLC, substandard, susceptibility

## Abstract

**Aims:**

In low‐ and middle‐income countries, antibiotic resistance is rising rapidly because of poor medication adherence, polypharmacy, and weak regulatory enforcement, while substandard and counterfeit medicines further accelerate resistance. Cefixime trihydrate, a broad‐spectrum third‐generation cephalosporin, is a widely used and frequently prescribed antibiotic in Bangladesh. This study evaluated quality attributes, including % drug content and % drug release, of eight commonly prescribed cefixime 200 mg tablet brands and compared them with the innovator‐equivalent brand.

**Methods:**

Chromatographic separation was performed on a reversed‐phase C18 column with UV detection at 282 nm (column temperature 30°C; run time 8 min), using methanol and 0.01 M KH_2_PO_4_ buffer (pH 4.5) at 1 mL/min. Dissolution studies were conducted using a USP Apparatus 2, and antimicrobial activity was assessed via the Kirby–Bauer disc diffusion method.

**Results:**

All nine brands met USP potency standards (90%–110%) and dissolution criteria (≥ 75% drug release within 45 min). A one‐way ANOVA with Dunnett′s test (*α* = 0.05, two‐tailed; critical value = 2.731) revealed significant differences between Tab. A, Tab. E, and Tab. F and the reference brand at 45 min. However, among local brands, seven out of eight (87.5%) complied with the FDA‐recommended difference factor (f1) and similarity (f2) factor criteria. Zone of inhibition studies showed good antimicrobial activity across most of the brands at 5 *μ*g disc load.

**Conclusion:**

Seven out of the eight local brands met the f1/f2 similarity criteria relative to the reference, indicating comparable in vitro dissolution behavior, whereas one brand (Tab. C) did not. These results emphasize the need for continuous quality surveillance to ensure therapeutic equivalence and combat antimicrobial resistance.

## 1. Introduction

Considering the worldwide increase in population, generic drugs were developed to make essential medicines more accessible to patients at lower prices. However, there is a lack of acceptance or distrust related to the quality and efficacy of generic medicines [1, 2]. Stringent regulatory frameworks have been established by developed countries to ensure the safety and efficacy of pharmaceutical products, including drugs. However, low‐ and middle‐income countries have often failed to ensure the quality of medicines because of technical and operational limitations of the regulatory authorities. Consequently, this enables the distribution of substandard and falsified medicines to go undetected [3–5]. According to the World Health Organization, approximately 1 in 10 medicines in low‐ and middle‐income countries failed quality control tests, suggesting that these products were either substandard or falsified [6]. Substandard and falsified medicines give rise to severe public health risks, causing poisoning, treatment failure, and worsening diseases. Moreover, substandard antibiotics result in subtherapeutic levels, allowing bacteria to survive, adapt, and develop resistance, thereby fueling the global antibiotic resistance crisis [7, 8]. The growing online sales of medicines through unauthorized platforms have worsened the issue, making it easier for falsified products to reach consumers.

Cefixime, a third‐generation cephalosporin, exerts its antibacterial effect by inhibiting bacterial cell wall synthesis. It binds to penicillin‐binding proteins (PBPs), particularly transpeptidases, disrupting the crosslinking of peptidoglycan layers in the bacterial cell wall. This weakens the cell wall, leading to cell lysis and bacterial death [9, 10]. Cefixime is indicated for the treatment of uncomplicated urinary tract infections (UTIs) caused by *Escherichia coli* and *Proteus mirabilis* and is also effective in managing otitis media associated with *Haemophilus influenzae*, *Moraxella catarrhalis*, and *Streptococcus* [11, 12]. Cefixime is also prescribed for pharyngitis and tonsillitis caused by *Streptococcus pyogenes*, as well as acute exacerbations of chronic bronchitis due to *Streptococcus pneumoniae* and *Haemophilus influenzae* [13]. Furthermore, it serves as a treatment option for uncomplicated cervical and urethral gonorrhea caused by *Neisseria gonorrhoeae*, including both penicillinase‐producing and nonpenicillinase‐producing strains [14].

In Bangladesh, there is a substantial lack of awareness of antimicrobial resistance among the general population. Moreover, a significant number of people have insufficient knowledge about medication adherence, persistence, and safe use. As a result, overuse and misuse of antibiotics contribute to the emergence of antibiotic‐resistant cases [15]. A systematic review by Ahmed et al. [16] reported that, across Bangladesh, *E. coli*, the most common UTI pathogen, showed high resistance to ampicillin (94.6%), amoxicillin (67.1%), ciprofloxacin (65.2%), and cotrimoxazole (72%), with similar trends in *Klebsiella*, *Pseudomonas*, and *Enterococcus* spp. Resistance to beta‐lactam antibiotics is notably high, indicating widespread production of extended‐spectrum beta‐lactamases (ESBLs), whereas carbapenems remain largely effective. Methicillin‐resistant *Staphylococcus aureus* (MRSA) is prevalent among *S. aureus* isolates. In contrast, *Salmonella* and *Shigella* spp. exhibit low resistance to key antibiotics [17–19]. Collectively, this evidence underscores the escalating threat of antibiotic resistance in Bangladesh.

Substandard and counterfeit antibiotics compromise patients′ recovery and increase the chance of antibacterial resistance. The low‐grade raw materials, insufficient doses, mislabeled content, and poor dissolution characteristics directly and indirectly contribute to treatment failure. As cefixime is a widely prescribed antibiotic in Bangladesh, this study is aimed at evaluating the quality of different cefixime trihydrate tablet brands using reversed‐phase high‐performance liquid chromatography (RP‐HPLC), dissolution testing, and microbiological assays to assess their therapeutic effectiveness and compliance with quality standards.

## 2. Materials and Methods

### 2.1. RP‐HPLC Materials and Methods

#### 2.1.1. Chemicals and Reagents

Standard cefixime trihydrate and analytical‐grade reagents, including potassium dihydrogen phosphate (KH_2_PO_4_), orthophosphoric acid (H_3_PO_4_), and sodium hydroxide (NaOH), were purchased from Sigma‐Aldrich (Merck), St. Louis, Missouri, United States. HPLC‐grade methanol and acetonitrile were obtained from Duksan Pure Chemicals Co., Ltd., Ansan, South Korea. The microorganisms *E. coli* (ATCC 25922) and *Vibrio cholerae* (ATCC 14033) were purchased from an authorized ATCC supplier. For bacterial growth and antimicrobial study, nutrient broth, nutrient agar, and Mueller–Hinton agar were obtained from HiMedia Laboratories Pvt. Ltd., Mumbai, India.

#### 2.1.2. Collection of Different Brands of Cefixime Trihydrate Tablets

A total of eight cefixime trihydrate tablet brands marketed in Bangladesh were collected from various models and community pharmacies in Dhaka and rural Gazipur. This strategy was adopted to ensure the inclusion of both urban and rural areas. The innovator‐equivalent cefixime trihydrate was sourced from a foreign country, accompanied by proper labeling, manufacturing license numbers, batch numbers, and expiration dates. All the collected brands indicate that the product contains 200 mg of cefixime trihydrate. During sample transport, standard procedures were followed to prevent degradation from environmental or other external factors. Samples collected from the local market were randomly coded A–H to prevent bias during analysis.

#### 2.1.3. Preparation of Buffer Solution

To prepare a 0.01 M KH_2_PO_4_ buffer solution, 1.390 g KH_2_PO_4_ salts were dissolved in a sufficient volume of Milli‐Q (Millipore, Bedford, Massachusetts, United States) water. The pH of the buffer solution was adjusted to 4.5 ± 0.01 by using a 10% (v/v) solution of H_3_PO_4_. Finally, the volume of the buffer solution was adjusted to 1.00 L. The buffer solution was then filtered through a 0.22 *μ*m hydrophobic polytetrafluoroethylene (PTFE) filter paper using a vacuum filtration apparatus, followed by sonication for 15 min.

#### 2.1.4. Preparation of Stock Solutions of Standard and Samples

A stock solution of standard cefixime trihydrate (500 *μ*g/mL) was prepared by dissolving 12.5 mg of cefixime trihydrate in 25 mL solvent containing buffer and methanol at a ratio of 3:1 and sonicated for 10 min to ensure complete dissolution. The standard stock solution was then filtered through a 0.22 *μ*m hydrophobic PTFE syringe filter obtained from Membrane Solution, United States. To obtain data for the standard curve, the standard stock solution (500 *μ*g/mL) was serially diluted to 200, 100, 50, 25, 10, and 5 *μ*g/mL. For sample preparation, 10 tablets from each brand were weighed to determine the average weight, then crushed and triturated into a fine powder using a mortar and pestle. The sample stock solution was prepared following the same procedure as the standard stock solution. The 500 *μ*g/mL sample stock solution was diluted to create a 50 *μ*g/mL working sample solution, which was analyzed by RP‐HPLC for both qualitative and quantitative purposes. Drug content was expressed as a percentage of the label claim. The same 10 tablets were weighed individually before crushing, and weight variation was expressed as the percentage deviation of each tablet from the mean tablet weight.

#### 2.1.5. Preparation of Dissolution Buffer and Assay Conditions

United States Pharmacopeia (USP) Apparatus 2 (Logan Instruments, United States; UDT‐804) was used to conduct the dissolution study. The concentration of the dissolution medium was 0.05 M phosphate buffer, and the pH was adjusted to 7.2 ± 0.01 using 10% sodium hydroxide solution. To simulate the physiological conditions in the human body, the temperature of the dissolution medium was maintained at 37^°^C ± 0.5^°^C with a paddle rotation speed of 100 rpm. For each dissolution study, six tablets from each brand were analyzed, and 5 mL samples were withdrawn at 0, 5, 15, 30, 45, and 60 min. An equal volume of fresh dissolution medium was added to maintain sink conditions and preserve the total volume in the vessel. The collected samples were then filtered using a 0.22 *μ*m PTFE syringe filter, diluted as required (50 *μ*g/mL), and analyzed using the validated HPLC method.

#### 2.1.6. RP‐HPLC Conditions

In this study, we used a Shimadzu LC‐20A series HPLC system, comprising two LC‐20AD solvent delivery pumps, a SIL‐20AC HT autosampler, a CTO‐20AC column oven, an SPD‐20A UV/VIS detector, and a CBM‐20A communications module. A reversed‐phase Shim‐pack GIST C18 column (250 × 4.6 mm, 5 *μ*m particle size) was used, and the temperature was maintained at 30°C. The run time was optimized to 8 min across multiple trials to achieve optimal chromatographic separation. Each sample was injected at a volume of 5 *μ*L. The mobile phase consisted of a buffer and methanol in a 3:1 ratio, delivered at a flow rate of 1 mL/min, with the detection wavelength (*λ*max) set at 282 nm. The method was developed and validated based on [20, 21]. Postrun analysis and chromatographic data were visualized and processed in Shimadzu LC Solution software.

#### 2.1.7. System Suitability

For the system suitability test, we replicated the injection of the same standard solution six times and confirmed that the theoretical plate was greater than 2000, the retention time of the samples was consistent across injections, and the deviation (%RSD) was less than 2%. The tailing factor for the samples was below 1.2, indicating that the peaks were symmetrical. Method precision was assessed by performing six replicate injections (*n* = 6) of the standard solution at three different concentration levels. Both repeatability (within‐day precision) and intermediate precision (between‐day) were satisfactory, with RSD values < 2%. Additionally, accuracy, robustness, specificity, and all other validation parameters were evaluated in accordance with ICH Q2(R2) guidelines [22] and are presented in the Table S1.

#### 2.1.8. Linearity

The linearity was validated using a 9‐point calibration curve of standard cefixime trihydrate. The calibration curves were linear over the ranges of 1, 2, 5, 10, 25, 50, 100, 200, and 500 *μ*g/mL. The correlation coefficient from regression analysis ranged from 0.9998 to 0.9999.

#### 2.1.9. Limit of Detection (LOD) and Limit of Quantitation (LOQ)

The LOD and LOQ were calculated using the standard deviation (SD) of the *y*‐intercepts from the regression analysis (*σ*) and the slope (*S*) of the calibration curve, according to the ICH Q2 equations of LOD = (3.3 × *σ*)/*S* and LOQ = (10 × *σ*)/*S* [22]. The LOD for this method was determined to be 0.033 *μ*g/mL, and the LOQ was 0.10 *μ*g/mL.

### 2.2. Materials and Methods for the Zone of Inhibition

#### 2.2.1. Preparation of Bacterial Growth Medium and Inoculum

To prepare nutrient agar media for microbial growth, 2.8 g of nutrient agar powder was dissolved in 100 mL of distilled water, and 45 mL of the prepared medium was dispensed into each Petri dish. The medium was used to culture microbes by the streak‐plate method, followed by overnight incubation at 37°C. To prepare nutrient broth, 1.2 g of nutrient broth powder was dissolved in distilled water, and the final volume was adjusted to 100 mL. Subsequently, 5 mL of the solution was dispensed into each test tube. The broth culture was prepared by transferring the tops of three or four colonies from the nutrient agar medium to the nutrient broth using a sterile loop and then incubating at 37°C overnight. Mueller–Hinton agar plates for disc diffusion assays were prepared by dissolving 19 g of agar in 500 mL of distilled water, and approximately 45 mL of the prepared medium was aseptically poured into each Petri dish. All the media were sterilized by autoclaving prior to use for microbial culture.

#### 2.2.2. Disc Preparation and Placement

The antibiotic disc solution for both cefixime trihydrate standard powder and the fine powder of marketed tablets was prepared by dissolving in water and a small amount of dimethyl sulfoxide (DMSO), followed by 10 min of sonication. The stock solution was prepared at 500 *μ*g/mL and further diluted to obtain working solutions from which the applied volumes delivered 10, 5, 0.5, 0.05, and 0.005 *μ*g cefixime per disc. The laboratory‐prepared 5 *μ*g disc was verified against a commercial 5 *μ*g cefixime disc (Biomaxima). Discs were prepared from Whatman Grade 3 filter paper using a clean disc punch to obtain discs approximately 6 mm in diameter. The prepared discs were then placed in a Petri dish, covered with foil, and sterilized in an autoclave at 121°C for 20 min at 15 psi. Sterile forceps were used to select an individual disc, and 10 *μ*L of antibiotic solution was dispensed at different concentrations. The discs were allowed to dry before being placed into the Petri dish. Bacterial culture broth from each of the bacterial strains was placed onto separate Petri dishes containing Mueller–Hinton agar. A sterile spreader was used to evenly distribute the bacteria across the entire surface of the agar. The antibiotic discs containing different cefixime loads (10, 5, 0.5, 0.05, and 0.005 *μ*g per disc) for the zone of inhibition study were placed with the commercially available standard disc (Biomaxima) and incubated overnight at 37°C. The zone of inhibition diameters for each drug were interpreted according to the guidelines provided by the Clinical and Laboratory Standards Institute (CLSI) [23] and the European Committee on Antimicrobial Susceptibility Testing (EUCAST) [24].

### 2.3. Statistical Analysis

One‐way ANOVA with post hoc analysis using Dunnett′s test (*α* = 0.05, two‐tailed; critical value = 2.731) was performed using GraphPad Prism. Fit factor analysis (f1 and f2) was conducted using DD Solver, a Microsoft Excel add‐in for dissolution data analysis. All other calculations were performed in Microsoft Excel.

## 3. Results

### 3.1. RP‐HPLC Results of Cefixime Trihydrate Tablets

All the samples were analyzed using RP‐HPLC with a UV/Vis detector. The HPLC data revealed (Figure 1) that all nine brands (eight local and one innovator‐equivalent foreign brand) complied with the USP acceptance criterion for tablets (90%–110% of the labeled amount). Representative chromatograms of the cefixime trihydrate standard (10–200 *μ*g/mL) and of the tablet samples (50 *μ*g/mL) are shown in Figures 2 and 3, where a single symmetrical peak was obtained at approximately 4 min retention time.

**Figure 1:**
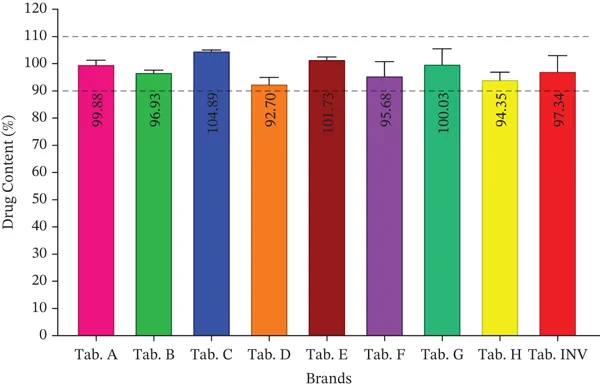
Comparison of the average % drug content with the labeled claim amount among different cefixime trihydrate tablet brands in Bangladesh. The dashed line indicates the USP acceptance range (90%–110%) for cefixime trihydrate tablets. Bars show the mean values; error bars show the standard deviation. Abbreviations: USP, United States Pharmacopeia; Tab., tablet brand; Tab. INV, innovator‐equivalent reference brand.

**Figure 2:**
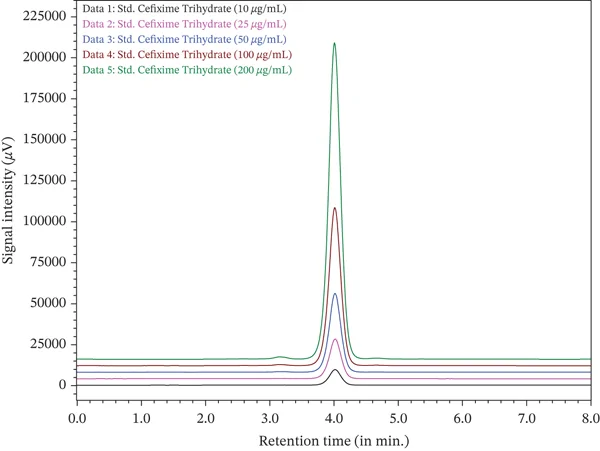
Superimposed RP‐HPLC chromatogram of standard cefixime trihydrate samples at different concentrations (10–200 *μ*g/mL). Abbreviation: RP‐HPLC, reversed‐phase high‐performance liquid chromatography.

**Figure 3:**
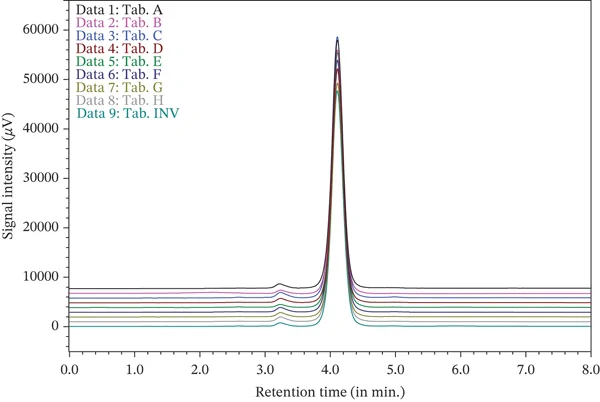
Superimposed RP‐HPLC chromatograms of different brands of cefixime trihydrate tablets at a concentration of 50 *μ*g/mL. Abbreviations: RP‐HPLC, reversed‐phase high‐performance liquid chromatography; Tab., tablet brand.

### 3.2. Dissolution Profile Analysis

#### 3.2.1. Dissolution Profile of Different Brands of Cefixime Trihydrate Tablets

According to the USP/NF specification, oral cefixime tablets should release not less than 75% (*Q*) of the labeled amount within 45 min using the USP Apparatus 2 [25]. According to USP General Chapter <711> Dissolution, harmonized across USP, PH. Eur., and JP for an immediate release tablet at Stage 1 (S1), each of the six tablets tested must release not less than 80% (*Q* + 5*%*) of the labeled amount of cefixime within 45 min. An additional six tablets are to be tested, and the average release from the 12 tablets should be at least 75% (*Q*), with no individual tablet releasing less than 60% (*Q* − 15*%*). Finally, an additional 12 tablets are to be tested if the S2 criteria are not met. The average release from all 24 tablets should be at least 75% (*Q*), with no more than two tablets releasing less than (*Q* − 15*%*) 60%, and no tablet releasing less than (*Q* − 25*%*) 50% [26]. The current study demonstrated (Table 1) that all the nine brands complied with the USP/NF dissolution test specification, following Stage 1 (S1) acceptance criteria of immediate release tablets. The drug release profile of different brand cefixime trihydrate is illustrated in Figure 4a (Tab. A, B, C, and D vs. Tab. INV) and Figure 4b (Tab. E, F, G, and H vs. Tab. INV). All brands except Tab. C released more than 70% of the labeled amount within 5 min, whereas Tab. C released only 21.19*%* ± 2.97*%* at 5 min and reached 84.26*%* ± 1.37*%* at 15 min (Table 1).

**Table 1:** Time‐dependent dissolution profile of different cefixime trihydrate tablet brands.

Time	Tab. A	Tab. B	Tab. C	Tab. D	Tab. E	Tab. F	Tab. G	Tab. H	Tab. INV
0 min	0.00 ± 0.00	0.00 ± 0.00	0.00 ± 0.00	0.00 ± 0.00	0.00 ± 0.00	0.00 ± 0.00	0.00 ± 0.00	0.00 ± 0.00	0.00 ± 0.00
5 min	83.54 ± 3.11	92.25 ± 4.88	21.19 ± 2.97	87.50 ± 10.26	96.98 ± 2.49	87.37 ± 3.92	90.17 ± 4.48	71.24 ± 12.74	93.93 ± 1.56
15 min	85.81 ± 3.50	95.20 ± 1.81	84.26 ± 1.37	91.71 ± 4.91	98.29 ± 1.84	87.87 ± 4.17	90.64 ± 4.41	91.66 ± 3.72	94.48 ± 2.32
30 min	88.46 ± 0.65	96.89 ± 1.51	93.57 ± 2.31	92.83 ± 4.88	99.60 ± 0.67	90.37 ± 1.36	94.25 ± 5.24	93.19 ± 2.62	94.88 ± 2.65
45 min	90.56 ± 0.67	97.11 ± 1.28	95.95 ± 1.29	94.69 ± 3.15	100.66 ± 1.09	91.01 ± 1.07	95.57 ± 4.33	93.37 ± 2.71	95.29 ± 2.88
60 min	93.78 ± 4.31	97.81 ± 1.27	102.36 ± 2.68	96.10 ± 3.16	101.59 ± 0.82	92.10 ± 0.54	96.55 ± 3.58	93.88 ± 3.22	96.36 ± 3.16

*Note:* Data presented as mean ± standard deviation of six tablets per brand (*n* = 6).

**Figure 4:**
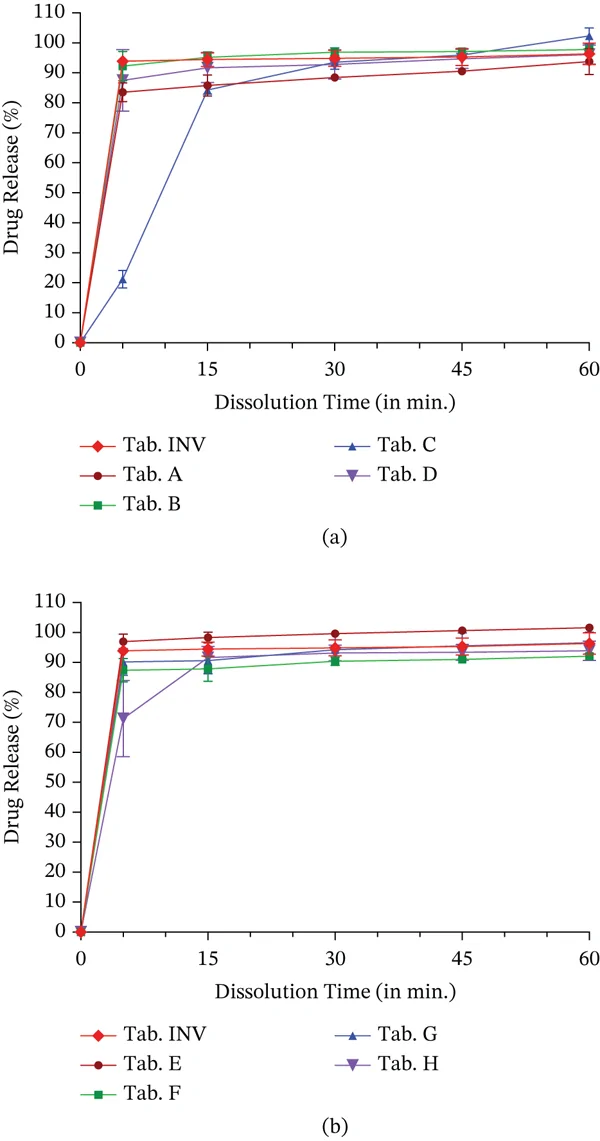
Comparative dissolution study of eight different brands of cefixime trihydrate tablets with the innovator‐equivalent brand (Tab. INV). (a) Comparison of four local brands (Tab. A, B, C, and D) with the innovator‐equivalent brand (Tab. INV). (b) Comparison of another four local brands (Tab. E, F, G, and H) with the innovator‐equivalent brand (Tab. INV). Data points represent mean ± standard deviation (*n* = 6). Abbreviations: Tab., tablet brand; Tab. INV, innovator‐equivalent reference brand.

#### 3.2.2. Fit Factor Analysis

Tablet dissolution profiles from different brands were compared with the innovator‐equivalent brand (Tab. INV) dissolution profile using the difference (f1) and similarity (f2) factors. Two dissolution profiles are considered similar and pharmacoequivalent if the difference factor (f1) is between 0 and 15 and the similarity factor (f2) is between 50 and 100, indicating equivalent performance of the test and reference products. The f1 value closer to 0 indicates very little difference between profiles, whereas an f2 value closer to 100 indicates stronger similarity [27]. The dissolution profile comparison of eight local cefixime trihydrate brands (Table 2) revealed that Tab. C failed both the f1 and f2 criteria, indicating significant differences from the reference (Tab. INV). The remaining seven local brands met the f1 and f2 criteria. Among these seven brands, Tab. H (f2 = 51.11) and, to a lesser extent, Tab. A (f2 = 59.10) lay close to the lower acceptance limit, indicating borderline similarity. As both products nevertheless released ≥ 85% of the labeled drug within the early sampling interval, these small differences are unlikely to be clinically meaningful but are reported transparently in line with regulatory guidance [27]. One‐way ANOVA of the percentage released at 45 min showed a significant overall difference among brands (Table 3), and Dunnett′s post hoc test identified Tab. A, E, and F as significantly different from Tab. INV (Table 4).

**Table 2:** Fit factor analysis data of different brand cefixime trihydrate tablets available in Bangladesh in comparison with the innovator‐equivalent brand.

Statistical parameter	Tab. A/INV	Tab. B/INV	Tab. C/INV	Tab. D/INV	Tab. E/INV	Tab. F/INV	Tab. G/INV	Tab. H/INV
f1	6.91	1.62	**19.15**	2.55	4.67	5.52	1.84	6.66
f2	59.10	87.60	**26.06**	75.04	68.60	65.08	80.72	51.11

*Note:* Profiles are considered similar when f1 = 0–15 and f2 = 50–100. Bold values indicate failure to meet the f1 and f2 criteria.

Abbreviations: f1, difference factor; f2, similarity factor; INV, innovator‐equivalent brand.

**Table 3:** Results of one‐way ANOVA for dissolution data of different tablet brands.

Time (min.)		SS	DF	MS	F (DFn, DFd)	*p*
45	Between brands	403.4	8	50.42	F (8, 45) = 22.61	*p* < 0.0001
	Within brands	100.4	45	2.23		
	Total	503.7	53			

Abbreviations: DF, degrees of freedom; MS, mean square; SS, sum of squares.

**Table 4:** Dunnett′s test for comparison of percentage of drug released at 45 min of different brands with the innovator‐equivalent reference brand at *α* = 0.05 (two‐tailed), critical value (*q*) = 2.731. Mean diff. = mean *%* released by Tab. INV minus that of the test brand.

Time (min.)	Dunnett′s multiple comparisons test	Mean diff.	Adjusted *p*	Significant?	*q*statistics
45	Tab. INV vs. Tab. A	4.652	< 0.0001	Yes	5.395
	Tab. INV vs. Tab. B	−1.46	0.4175	No	1.693
	Tab. INV vs. Tab. C	−0.6367	0.9741	No	0.7384
	Tab. INV vs. Tab. D	0.56	0.9883	No	0.6495
	Tab. INV vs. Tab. E	−4.83	< 0.0001	Yes	5.602
	Tab. INV vs. Tab. F	4.26	< 0.0001	Yes	4.941
	Tab. INV vs. Tab. G	0.3083	0.9995	No	0.3576
	Tab. INV vs. Tab. H	1.925	0.1605	No	2.233

#### 3.2.3. Drug Release Kinetics

Data obtained from the in vitro dissolution studies were analyzed for drug release kinetics using DD Solver, a Microsoft Excel add‐in for dissolution data analysis. Several kinetic models, including zero‐order, first‐order, Higuchi, Hixson–Crowell, Korsmeyer–Peppas, Weibull, probit, and logistic, were applied to the dissolution data. The goodness‐of‐fit for each model was assessed using the coefficient of determination (*R* ^2^), Akaike information criterion (AIC), and model selection criterion (MSC). A model with a lower AIC value is considered the best fit; an MSC value greater than 2 or 3 indicates a good fit, whereas an *R* ^2^ value closer to 1 suggests a stronger correlation between the model and the experimental data [28]. Table 5 demonstrates that none of the nine brands followed the zero‐order, Higuchi, or Hixson–Crowell kinetic models. In contrast, all brands exhibited good fit with the first‐order, Weibull, logistic, and probit models. Tab. C showed poor fit with the Korsmeyer–Peppas model, whereas all other brands complied with it.

**Table 5:** Drug release kinetic model fitting of different brands of cefixime trihydrate tablets.

Kinetic models	Parameters	Tab. A	Tab. B	Tab. C	Tab. D	Tab. E	Tab. F	Tab. G	Tab. H	Tab. INV
Zero‐order	*R* ^2^	−0.5171	−0.6406	0.5486	−0.5749	−0.6539	−0.6339	−0.5943	−0.3340	−0.6982
	AIC	57.2520	58.6456	52.2927	58.0027	59.1276	57.8303	58.1793	56.7943	58.7313
	MSC	−2.4945	−2.6073	−0.1417	−2.5495	−2.6201	−2.6037	−2.5689	−2.1697	−2.6524
	k0	2.075	2.219	2.160	2.159	2.298	2.079	2.174	2.120	2.185

First‐order	*R* ^2^	0.9319	0.9941	0.9647	0.9777	0.9993	0.9438	0.9794	0.9769	0.9878
	AIC	38.6346	24.8972	36.9946	32.4598	12.7806	37.6070	32.0774	32.4519	29.1181
	MSC	0.6084	3.0175	2.4080	1.7077	5.1044	0.7669	1.7814	1.8873	2.2831
	k1	0.345	0.510	0.087	0.409	0.700	0.404	0.458	0.238	0.559

Higuchi	*R* ^2^	0.4057	0.3281	0.8780	0.3722	0.3151	0.3285	0.3557	0.5383	0.2825
	AIC	51.6293	53.2895	44.4415	52.4842	53.8380	52.4950	52.7437	50.4281	53.5619
	MSC	−1.5574	−1.7146	1.1669	−1.6297	−1.7385	−1.7145	−1.6630	−1.1087	−1.7909
	kH	15.081	16.224	14.985	15.735	16.808	15.191	15.857	15.343	16.008

Korsmeyer–Peppas	*R* ^2^	0.9992	0.9999	0.8981	0.9999	0.9999	0.9998	0.9995	0.9872	0.9999
	AIC	14.0485	−0.7773	45.3602	2.7469	−0.4590	6.9465	11.7039	30.9182	0.5510
	MSC	4.7060	7.2965	1.0137	6.6598	7.3110	5.8770	5.1770	2.1429	7.0443
	kKP	76.945	89.104	22.730	82.705	93.866	83.774	85.118	65.012	92.388
	*n*	0.044	0.023	0.386	0.036	0.018	0.022	0.030	0.098	0.009

Hixson–Crowell	*R* ^2^	0.5143	0.4664	0.9710	0.5050	0.4377	0.4550	0.4827	0.6921	0.4217
	AIC	50.4179	51.9066	35.8282	51.0584	52.6543	51.2428	51.4265	47.9977	52.2682
	MSC	−1.3555	−1.4841	2.6024	−1.3921	−1.5412	−1.5058	−1.4434	−0.7036	−1.5752
	KHC	0.025	0.026	0.023	0.025	0.026	0.025	0.025	0.025	0.026

Weibull	*R* ^2^	0.9988	1.0000	0.9936	0.9998	0.9995	0.9997	0.9993	0.9940	0.9999
	AIC	16.4175	−9.4522	28.7321	5.1058	12.5840	8.2037	14.1018	26.3745	1.3655
	MSC	4.3112	8.7424	3.7851	6.2667	5.1371	5.6674	4.7773	2.9002	6.9085
	*α*	0.719	0.505	81.152	0.624	0.464	0.562	0.558	1.427	0.393
	*β*	0.143	0.159	1.846	0.160	0.291	0.080	0.138	0.392	0.053

Logistic	*R* ^2^	0.9986	1.0000	0.9970	0.9998	0.9994	0.9997	0.9992	0.9964	0.9999
	AIC	17.3285	−8.3791	24.1688	6.4836	13.1054	8.7625	14.9398	23.3271	1.6498
	MSC	4.1594	8.5635	4.5457	6.0370	5.0502	5.5743	4.6377	3.4081	6.8612
	*α*	1.030	1.654	−5.484	1.241	1.299	1.567	1.522	−0.555	2.443
	*β*	0.763	1.167	6.005	0.990	3.045	0.452	0.869	2.163	0.375

Probit	*R* ^2^	0.9987	1.0000	0.9958	0.9998	0.9995	0.9997	0.9992	0.9954	0.9999
	AIC	16.9173	−9.3796	26.2437	5.7746	12.8258	8.4968	14.5243	24.8051	1.5007
	MSC	4.2279	8.7303	4.1998	6.1552	5.0968	5.6186	4.7069	3.1618	6.8860
	*α*	0.654	1.047	−3.297	0.799	1.058	0.952	0.944	−0.198	1.410
	*β*	0.410	0.535	3.601	0.495	1.142	0.236	0.430	1.143	0.175

*Note:* Negative *R* ^2^ values indicate a fit poorer than the mean of the data.

Abbreviations: *R* ^2^, coefficient of determination; AIC, Akaike information criterion; MSC, model selection criterion.

### 3.3. Microbiological Assay

The antibacterial activity of nine marketed cefixime trihydrate tablet brands and the standard reference was assessed against *E. coli* (ATCC 25922) and *V. cholerae* (ATCC 14033) using the Kirby–Bauer disc diffusion method. The zone diameter interpretation for *E. coli* followed CLSI and EUCAST breakpoints, whereas for *V. cholerae*, Enterobacteriaceae breakpoints were used as reference. A standard 5 *μ*g cefixime disc (Biomaxima) matched our laboratory‐prepared 5 *μ*g disc; the lower, substandard loads were exploratory, and their reduced zones reflect a concentration effect, not resistance or poor quality. As all brands share the same active ingredient, disc diffusion confirmed antibacterial activity rather than discriminating equivalence. Figure 5 demonstrates that both *E. coli* and *V. cholerae* showed no measurable inhibition at the 0.005 *μ*g antibiotic disc load. At 0.05 *μ*g, no inhibition zone was observed against *V. cholerae*, whereas in the case of *E. coli*, all brands except Tab. C, Tab. F, and the standard formulation displayed resistance. For 0.5 *μ*g concentration, only Tab. E exhibited an intermediate susceptibility pattern, similar to the standard, against *V. cholerae*. Conversely, for *E. coli*, Tab. B demonstrated an intermediate response, whereas Tab. C, F, H, and the standard were susceptible; the remaining brands showed resistance. At 5 *μ*g, most brands were susceptible; the exception was Tab. A (resistant against both organisms), Tab. G (intermediate against *E. coli* and resistant against *V. cholerae*), and Tab. B (resistant against *V. cholerae*), whereas at 10 *μ*g all brands were susceptible.

**Figure 5:**
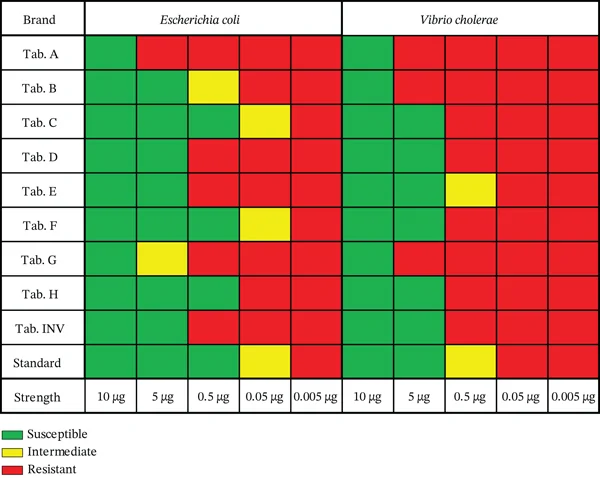
Heat map indicating zone of inhibition of cefixime trihydrate tablets and standard at different concentrations against *E. coli* and *V. cholerae*. Green color indicates susceptibility, yellow color indicates intermediate susceptibility, and red color indicates resistance.

## 4. Discussion

It is challenging to determine the relative impact of counterfeit and substandard medicines, as comprehensive data on their prevalence and consequences remain limited. Additionally, there is a lack of awareness among patients and healthcare professionals about counterfeit and substandard medicines and their effective interventions. Further, the lack of adequate resources to establish efficient postmarket assessment systems exacerbates the problem. Because the adverse effects of substandard medicines on both individuals and public health often go unreported, there is little motivation for timely intervention [29, 30].

Conducting postmarket quality assessments of marketed drugs is crucial for ensuring their safety, efficacy, and regulatory compliance. These assessments help detect substandard or counterfeit formulations, ensuring that drugs meet pharmacopeial standards for active pharmaceutical ingredient (API) content, dissolution, and impurity levels. The detection and monitoring of impurities in marketed drugs remain underexplored, highlighting a critical gap in pharmaceutical research. By identifying inconsistencies, postmarket surveillance enables regulatory bodies to monitor drug quality, enforce stricter policies, and mitigate potential health risks. Additionally, it safeguards therapeutic outcomes, preventing treatment failures and reducing the risk of antimicrobial resistance. This process also plays a crucial role in identifying adverse effects, monitoring real‐world drug performance, and ensuring ongoing compliance with regulatory standards. By supporting risk management, informing clinical practice, and strengthening public trust, postmarket surveillance ultimately contributes to better patient outcomes and the integrity of the healthcare system.

As part of the quality control process, it is essential to ensure that generic drugs are both pharmaceutically equivalent and bioequivalent to their innovator counterparts. Equally important is verifying that data obtained from dissolution tests and other quality control studies comply with regulatory acceptance guidelines. In this context, (Figure 1) our study revealed that all the brands (nine out of nine) met the official USP potency limit (90%–110%) for cefixime trihydrate content relative to the labeled amount (200 mg). Figures 2 and 3 illustrate the HPLC chromatograms of cefixime trihydrate, where Figure 2 depicts standards at 10–200 *μ*g/mL and Figure 3 shows marketed tablet brands at 50 *μ*g/mL.

Average tablet weights (Table 6) ranged from 327.75 mg to 510.75 mg, reflecting differences in formulation among brands. Within each brand, SD values were low (± 3–8 mg), indicating consistent weight uniformity. Most brands showed weight variation within ± 3%, meeting pharmacopeial limits (± 5% for tablets > 250 mg). Tab. A, B, C, E, F, G, and H had acceptable variation. Brand D showed a higher deviation (+5.51%), suggesting possible manufacturing inconsistencies due to variations in granulation, compression force, or blend uniformity during tablet production.

**Table 6:** Weight variation in different marketed cefixime trihydrate tablet brands.

Brands	Average weight ± SD (in mg)	Weight variation range (%)
Tab. A	454.68 ± 3.83	−1.80 to +0.89
Tab. B	456.44 ± 4.34	−1.56 to +1.20
Tab. C	451.25 ± 3.16	−1.47 to +0.85
Tab. D	468.75 ± 12.7	−2.66 to +5.51
Tab. E	381.74 ± 4.74	−1.58 to +1.85
Tab. F	448.39 ± 5.11	−2.41 to +1.12
Tab. G	404.53 ± 3.41	−1.12 to +1.63
Tab. H	510.75 ± 8.07	−1.24 to +3.75
Tab. INV	327.75 ± 4.44	−2.15 to +2.33

*Note:* Data presented as mean ± standard deviation of 10 tablets per brand (*n* = 10).

Table 1 shows that all brands released increasing amounts of cefixime over time. Most brands achieved ≥ 75% release within 45 min, meeting USP dissolution criteria, whereas Tab. C showed slower release. Table 2 shows how dissolution profiles are compared for similarity and dissimilarity. Here, Tab. C shows a significant deviation from the reference brand in both f1 (dissimilarity) and f2 (similarity). The marked dissimilarity observed in Tab. C suggests potential formulation inconsistencies, possibly due to variations in excipient composition, compression force, or coating uniformity. This indicates subtle formulation or process‐related variations not detected by routine compendial tests, highlighting that meeting pharmacopeial specifications alone may not guarantee interchangeability, emphasizing the value of dissolution profile comparison in postmarket quality evaluation. To identify differences between brands, one‐way ANOVA and Dunnett′s test were used to compare local brands with Tab. INV at the 0.05 level (two‐tailed). Results are shown in Tables 3 and 4. Values above the critical value (2.731) indicate a significant difference, whereas values below the critical value indicate no significant difference. Brands Tab. A, Tab. E, and Tab. F showed significant difference at 45 min of dissolution. For these three brands, the mean differences from the reference (Dunnett‐adjusted 95% confidence intervals; pooled SD = 1.49*%*) were +4.65% (2.30–7.00) for Tab. A, −4.83% (−7.18 to −2.48) for Tab. E, and +4.26% (1.91–6.61) for Tab. F. The corresponding standardized effect sizes (Cohen′s *d*) were 3.1, 3.2, and 2.9; these large standardized values reflect the very low within‐brand variability rather than large absolute differences, as every deviation was < 5% at a stage where all brands had released > 90% of the labeled drug. ANOVA and Dunnett′s test reveal statistical differences rather than pharmaceutical equivalency. Statistical equivalence indicates no significant difference between brands at the 0.05 level, whereas pharmaceutical equivalence indicates dissolution patterns within specification [31].

To determine drug release kinetics, various model‐dependent approaches were analyzed (Table 5). The analysis of cefixime trihydrate tablets across various brands reveals that the first‐order, Korsmeyer–Peppas, Weibull, probit, and logistic models provide the best fit for most brands. Only Tab. C showed poor fit with the Korsmeyer–Peppas. None of the tablets from the nine different brands followed zero‐order, Higuchi, or Hixson–Crowell kinetic models, indicating that drug release from these tablets did not occur in a concentration‐independent (zero‐order), diffusion‐controlled (Higuchi), or erosion‐controlled (Hixson–Crowell) manner. Lower *R* ^2^ values and negative MSC values reflect poor fit in these models. Because all formulations released most of their drug within the first sampling interval, these models are best read as empirical descriptors of comparative release rate rather than evidence of a specific mechanism. The good first‐order and Weibull fits and poor zero‐order, Higuchi and Hixson–Crowell fits, are consistent with rapidly dissolving immediate‐release tablets.

From the antimicrobial susceptibility test (Figure 5), it clearly indicates that *E. coli* and *V. cholerae* showed reduced or no inhibition at the lower disc loads (0.005–0.05 *μ*g). At 0.5 *μ*g, a few brands showed intermediate susceptibility to both *E. coli* and *V. cholerae*, whereas at higher concentrations (5–10 *μ*g), both bacteria demonstrated progressively greater antibacterial activity. In this study, the measurable zone of inhibition for most brands appeared at 0.5–5 *μ*g disc loads, indicating that higher antibiotic concentrations are required to achieve effective inhibition of both bacterial species.

This study provides an overview of the quality of marketed cefixime trihydrate brands in Bangladesh, though some limitations apply: the tablet brands sampled from Dhaka and Gazipur and the single batch tested per brand limit national and batch‐to‐batch generalizability; rapid dissolution weakens f1/f2 discrimination; and the microbiological assay only confirmed antibacterial activity. Continuous postmarketing surveillance and future multibatch studies, including impurity profiling, are therefore essential to ensure ongoing product quality.

## 5. Conclusion

In this study, we evaluated eight different cefixime trihydrate tablet brands available in Bangladesh against the innovator‐equivalent brand. The study showed that all brands met the USP potency standards and complied with the USP dissolution test criteria (drug release not less than 75% within 45 min). However, 87.5% (seven out of the eight local brands) comply with the FDA‐recommended difference (f1) and similarity (f2) factors, indicating similar dissolution profiles with the reference product. One brand (Tab. C) did not meet these similarity criteria, underscoring that compliance with compendial assay limits alone does not guarantee equivalent in vitro dissolution performance and supporting the need for continued, multibatch postmarket surveillance. The zone of inhibition study revealed good antimicrobial sensitivity for most of the cefixime trihydrate brands at the standard disc content, whereas the absence of inhibition at the lowest disc loads reflected an expected concentration‐dependent effect rather than bacterial resistance or poor product quality. Ensuring the quality and therapeutic equivalence of marketed antibiotics is crucial to minimizing treatment failure and limiting the emergence of antimicrobial resistance. Such surveillance also supports rational antibiotic use and strengthens healthcare outcomes, particularly in resource‐limited settings.

## Author Contributions

Conceptualization: M.H.S. Methodology: A.I., S.A.S., S.A., A.N., M.M., Z.F.F., A.A., and M.H.S. Software: F.K. Validation: F.K. and M.H.S. Formal analysis: A.I., S.A.S., S.A., A.N., F.K., and M.H.S. Investigation: A.I., S.A.S., S.A., A.N., M.M., Z.F.F., A.A., F.K., and M.H.S. Resources: M.H.S. Data curation: F.K. and M.H.S. Writing—original draft preparation: A.I., S.A.S., and F.K. Writing—review and editing: M.H.S. Visualization: A.I. and S.A.S. Supervision: M.H.S. Project administration: M.H.S.

## Funding

No funding was received for this manuscript.

## Conflicts of Interest

The authors declare no conflicts of interest.

## Supporting information


**Supporting Information** Additional supporting information can be found online in the Supporting Information section. Table S1: Validation Parameters of the RP‐HPLC method for the assay of cefixime trihydrate in tablets.

## Data Availability

The data that support the findings of this study are available from the corresponding author upon reasonable request.
