# Supplementary material for: Comparative Quality Evaluation of Marketed Cefixime Trihydrate Tablet Brands Available in Bangladesh
Source: ScientificWorldJournal. 2026 Oct 26;2026:4894162. doi: 10.1155/tswj/4894162 (PMC13699413; doi:10.1155/tswj/4894162)
Supplement: Supplementary file 1 [file TSWJ-2026-4894162-s001.docx]

**Supplementary Table S1:** Validation Parameters of the RP-HPLC method for the assay of cefixime trihydrate in tablets.

| **Validation Parameters** | **Results** | **%RSD** |
| --- | --- | --- |
| **Precision** | | |
| Repeatability | 99.88 ± 0.61 | 0.61% |
| Intraday Precision | 99.76 ± 0.56 | 0.56% |
| Interday Precision | 98.85 ± 1.08 | 1.09% |
| **Accuracy** | | |
| Spiked Level 80% | 99.43 ± 0.52 | 0.52% |
| Spiked Level 100% | 100.21 ± 0.47 | 0.47% |
| Spiked Level 120% | 99.79 ± 0.63 | 0.63% |
| **Robustness** | | |
| Flow Rate Change  (0.8-1.2) mL/min. | 99.50 ± 0.88 | 0.88% |
| Buffer pH Change  (4.4-4.6) | 100.11 ± 0.31 | 0.31% |
| Column Temperature  (25-35) °C | 99.45 ± 0.73 | 0.73% |
| Detection Wavelength | 99.64 ± 0.25 | 0.25% |
| **Linearity** | | |
| Coefficient of Determination R^2^ | 0.9998 | - |
| Straight Line Equation | 15567x + 14397 | - |
| **LOD** | 0.033 µg/mL | - |
| **LOQ** | 0.10 µg/mL | - |
| **Specificity** | Specific | - |

***LOD*** *= Limit of detection;* ***LOQ*** *= Limit of quantification;* ***%RSD*** *= percentage relative standard deviation.*
